# Prevalence of Pain and Its Relationship with Age and Sex among Patients in Saudi Arabia

**DOI:** 10.3390/jcm13010133

**Published:** 2023-12-26

**Authors:** Ali M. Alshami

**Affiliations:** Department of Physical Therapy, College of Applied Medical Sciences, Imam Abdulrahman Bin Faisal University, P.O. Box 2435, Dammam 31441, Saudi Arabia; alshami@iau.edu.sa; Tel.: +966-013-3331263

**Keywords:** association, musculoskeletal, physical therapy

## Abstract

Background: Studies investigating the prevalence of patients with pain referred for physical therapy in Saudi Arabia are scarce. This study aimed to estimate the period prevalence of pain that led to referrals for physical therapy and to evaluate the association between pain and patient age and sex. Methods: This retrospective study used data from the electronic health record system of a hospital for adult patients referred for physical therapy. Results: In total, 7426 (26.0%) patients (mean (±SD) age, 51.4 ± 15.0 years) experienced pain, the majority of whom were female (65.8%). The back (30.7%) was the most commonly reported pain region, followed by the neck (13.2%), shoulders (12.1%), and knees (11.8%). The referring physician(s) identified pain in a specific body region in 5894 of the 7426 (79.4%) patients. A moderate correlation was found between sex and pain region (Cramer’s V = 0.151, *p* < 0.001) and between age group and pain region (Cramer’s V = 0.10, *p* < 0.001). Conclusions: Pain was prevalent among adult patients referred for physical therapy and was moderately associated with sex and age. Further research examining the prevalence of pain and its risk factors in a larger, representative sample of the population is warranted.

## 1. Introduction

The International Association for the Study of Pain recently revised the definition of pain to be “An unpleasant sensory and emotional experience associated with, or resembling that associated with, actual or potential tissue damage” [1]. Between 2009 and 2021, the prevalence of pain rose from 26.3% to 32.1%, according to a new study using nationally representative data from 146 countries (N = 1.6 million respondents). This concerning trend was observed in both higher- and lower-income countries [2]. Pain, the leading cause of both seeking medical care and disability globally, incurs significant economic costs. Unlike acute pain, which serves a vital survival function, chronic pain is best understood as a disease itself [3].

The prevalence of chronic pain has been estimated to be from 11% to 40% in the United States and 43.5% in the Untied Kingdom (UK), with an annual incidence of 8.3% in the UK [3]. Management of chronic pain requires a multifaceted approach, including interventions such as encouraging activity despite pain and fostering psychological well-being through pain acceptance and optimism [3]. Musculoskeletal pain is the most prevalent type of chronic pain. This category includes conditions like low back pain (LBP), neck pain, and arthritis pain. Other common types of chronic pain include neuropathic pain, functional pain syndromes, chronic pain after surgery, complex regional pain syndrome, and cancer pain [4]. LBP remains the most significant contributor to disability burden globally. In 2020, an estimated 619 million people lived with LBP, a staggering number demonstrating its widespread impact. Additionally, LBP caused an estimated 69 million years lived with disability (YLDs), representing 7.7% of all YLDs globally [5].

Research on the prevalence of pain in the Middle East remains limited. However, a few studies shed light on the prevalence of specific pain syndromes. For instance, a recent study estimated the age-standardized point prevalence of LBP in the Middle East and North Africa region to be 7.66%, highlighting the significant burden of this condition on millions of individuals [6]. Few studies have investigated the prevalence of pain in Saudi Arabia. Moreover, most of these studies were limited only to certain disorders, such as LBP [7,8], presented as secondary analyses, derived from work-related musculoskeletal problems [8], or conducted on healthcare professionals [9].

Studies investigating the prevalence and features of patients with pain syndromes referred for physical therapy are lacking. Collecting information regarding the prevalence of pain helps policymakers allocate resources and improve the quality of care for pain, related disabilities, and psychosocial well-being [10,11]. As such, the aim of the present study was to estimate the period prevalence of pain that led to referrals for physical therapy and to evaluate the association between pain region(s) and patient age and sex.

## 2. Materials and Methods

Ethics approval for this study was obtained from the Institutional Research Board of the Imam Abdulrahman Bin Faisal University, Saudi Arabia (IRB-2023-03-503, date: 15 November 2023). This retrospective study was conducted at the Department of Physical Therapy, King Fahd Hospital of the University (KFHU), Eastern Province, Saudi Arabia. This hospital has >600 beds and is comprised of several inpatient and outpatient departments and clinics. The Department of Physical Therapy provides care to patients referred by physicians inside and outside the hospital [10]. Physical therapists, physicians, and other healthcare practitioners often work in multi-disciplinary teams to manage patients experiencing pain. In Saudi Arabia, patients pursuing physical therapy usually require referral from a physician [10].

All patients at KFHU, regardless of age, can be electronically referred to physical therapy through the hospital’s electronic health record (EHR) system (Harris Flex version 6.4.2.20, Harris Healthcare, Niagara Falls, NY, USA). Physicians should include an accurate diagnosis in the referral along with any necessary precautions and contraindications. Referrals are appropriate for patients experiencing pain or with any of the following conditions: orthopedic and musculoskeletal problems (bone fractures, joint pain, muscle weakness, and limitations in range of motion); neurological disorders (stroke, multiple sclerosis, and Parkinson’s disease); post-surgical rehabilitation (to help patients regain strength, flexibility, and function after surgery); balance and gait disturbances (to improve stability and prevent falls); and pre-surgical preparation (to help prepare patients for surgery by improving their strength and endurance).

Data from all new adult patients, who were electronically referred to the Department of Physical Therapy, were retrieved from the EHR system and reviewed. The retrieved data were limited to patient nationality, sex, age, referral facility/service, care type, and diagnoses of physicians and physical therapists. The data collected spanned a period of 5 years—more specifically, from 1 January 2017 to 31 December 2022. Patient records that included initial and progress notes from physicians and physical therapists were not retrieved.

All data retrieved from the EHR system were exported to Excel^®^ version 2021 for Microsoft 365 (Microsoft Corporation, Redmond, Washington, DC, USA). This widely used spreadsheet software provides a familiar and flexible environment for data analysis and visualization. By exporting the data to Excel, we were able to perform basic and advanced data cleaning and manipulation. After initial correction of spelling errors to ensure patient inclusion and minimize missing data, we employed Launch Power Query Editor in Excel. This feature provides access to the powerful Power Query tool for data transformation and cleansing [12]. We then applied advanced filtering in Excel using two columns: “physician’s diagnosis” and “impression of physical therapist”. This filtering aimed to identify all possible keywords expressing “pain”. These keywords included “pain”, “algia”, “LBP”, “lumbago”, and “ache”. Subsequently, we utilized the same approach to filter and categorize patients with pain according to body regions. This involved identifying all possible keywords for each region, including head/face, neck/cervic*, chest, abdom*, shoulder, elbow, wrist/hand, LBP/back/lumb*, pelv*, hip, knee, and ankle/foot.

### Data Analysis

Data were analyzed using SPSS version 27.0 (IBM Corporation, Armonk, NY, USA) for Windows (Microsoft Corporation, Redmond, WA, USA). Descriptive statistics, frequencies, and percentages were calculated to determine prevalence. An independent *t*-test was used to compare male and female patients. The associations between age group (ordinal) and leading painful regions (nominal) and between sex (nominal) and leading pain regions were examined using chi-squared and Cramer’s V tests. Cramer’s V-test values were interpreted as no or very weak (>0), weak (>0.05), moderate (>0.10), strong (>0.15), or very strong (>0.25) [13]. Differences in age (continuous) among the leading pain regions were evaluated using one-way analysis of variance (i.e., “ANOVA”), followed by the Bonferroni test (with post hoc correction). Differences with *p* < 0.05 were considered to be statically significant.

## 3. Results

From 1 January 2017 to 31 December 2022, a total of 28,621 adult patients were referred for physical therapy. Of these, 26.0% (*n* = 7426) reported experiencing pain at different body sites. The remaining 74.0% (*n* = 21,195) had disorders other than pain. Patients generally shared similar characteristics regardless of pain status. Most were over 50 years old, with a slight majority being female, particularly among those experiencing pain. Additionally, the majority of patients were Saudi nationals and were referred to outpatient clinics, with orthopedics being the most common referring service (Table 1).

The pain regions reported by the 7426 referred patients are summarized in Table 2. The back (30.7%) was the most common pain region, followed by the neck (13.2%), shoulders (12.1%), and knees (11.8%). However, in 1531 (20.6%) patients, the referring physician(s) did not identify the specific body region affected by pain; as such, the results of patients with specific regions identified (*n* = 5894) will be discussed. Of 5894 patients with pain affecting specific body regions, females (*n* = 4051 (68.7%)) outnumbered males (*n* = 1843 (31.3%)). Female patients were older (52.9 ± 14.1 years) than male patients (48.5 ± 16.1 years) (mean difference, 4.4 years (95% confidence interval (CI) −5.199 to −3.577); t = −10.601; degrees of freedom (df) = 589; *p* < 0.001).

Among the 5894 patients, 766 (13%) experienced pain in two or more body regions. These patients had a mean age of 53.4 years (SD 14.9) and were predominantly women (191 female vs. 575 male). Additionally, a majority were Saudi citizens (673 vs. 93 non-Saudis). The pain distribution in patients with multisite pain was as follows: 2 regions (622 patients (81.2%)); 3 regions (124 patients (16.2%)); 4 regions (16 patients (2.1%)); and 5 regions (4 patients (0.5%)). The most common painful regions were back (*n* = 380), knee (*n* = 344), neck (*n* = 311), shoulder (*n* = 299), and ankle/foot (*n* = 108). Patients with multisite pain were all referred to outpatient physical therapy, either during their initial evaluation or on separate dates throughout the study period.

**Figure 1:**
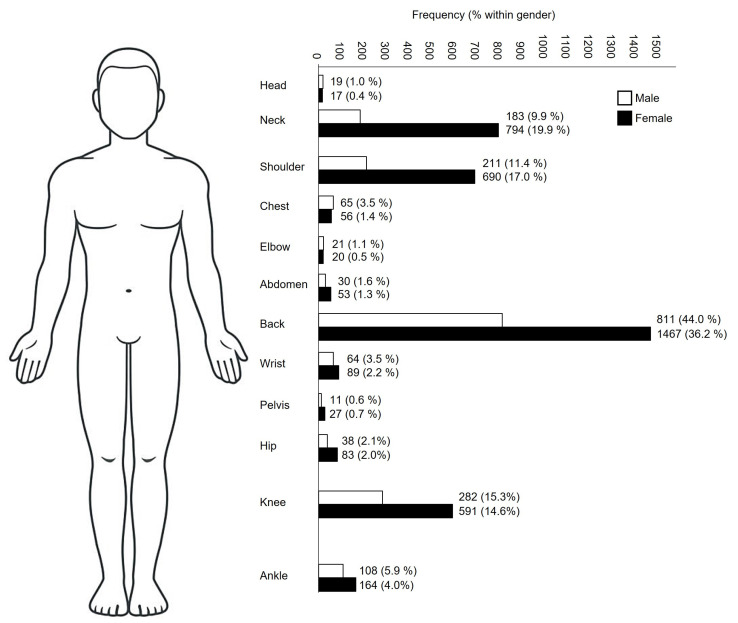
Frequency and percentage of adult patients who were referred with pain in specific body regions (*n* = 5894). Body chart from https://www.istockphoto.com, accessed on 16 November 2023.

The most common painful body regions included the back, neck, shoulders, and knees. The characteristics of patients reporting pain in the four most commonly pain regions (>10% of the total referred patients with pain) are summarized in Figure 1 and Table 3. Notably, the proportion of patients admitted to inpatient care was higher among individuals suffering from knee (*n* = 119) or back (*n* = 208) pain compared to those experiencing neck (*n* = 12) or shoulder (*n* = 14) pain.

A moderate relationship was observed between age and pain region(s) (Cramer’s V = 0.10, *p* < 0.001). ANOVA revealed that age had a significant effect on the pain region (df = 3, F = 22.006, *p* < 0.001). Older patients exhibited more prevalent pain in the shoulder than in the neck (*p* < 0.001 (95% CI −4.975 to −1.466)), back (*p* < 0.001 (95% CI −6.049 to −3.058)), and knee (*p* < 0.001 (95% CI 0.814 to 4.422)) and more pain in the knee compared with the back (*p* = 0.004 (95% CI 0.424 to 3.448)). Patient sex was moderately associated with pain region (Cramer’s V = 0.151, *p* < 0.001). Patients 50–69 years of age exhibited more prevalent pain in all four most common body regions (back, *n* = 1046 (45.9%); neck, *n* = 501 (51.3%); shoulder, *n* = 473 (52.5%); and knee, *n* = 372 (42.6%)).

## 4. Discussion

The present study aimed to determine the prevalence of pain among adult patients referred for physical therapy in the Eastern Province of Saudi Arabia over a 5-year period. Approximately 26.0% (*n* = 7426) of the referred patients experienced pain in different regions of the body. The four most common pain regions were the back, neck, shoulder, and knees. Moderate associations were found between age group and pain regions and between sex and pain regions. Shoulder pain was more prevalent in older patients than neck, back, or knee pain. They also reported a higher prevalence of pain in the knee than in the back.

While most studies investigating pain prevalence, both locally and globally, have focused specifically on chronic pain [3,14], interpreting comparisons with their reported prevalence rates becomes challenging in our study. This difficulty arises from the potential inclusion of patients seeking physical therapy for acute pain syndromes. Studies investigating the prevalence of pain among the general population in Saudi Arabia are scarce. Cross-sectional studies have found that the prevalence of self-reported chronic pain was 47.5% in the Makkah region [14], 19% in Al Kharj City [11], and 46.4% in populations from 5 regions, including the Eastern Province [15]. The prevalence of chronic pain in developing countries is 28% in the Middle East and North Africa [16] and 56% in Kuwait, a neighboring country [17]. In developed countries, the prevalence of chronic pain is estimated to be from 11% to 40% in the United States, 43.5% in the UK [3], and 31.7% in France [18]. The discrepancy in the prevalence of pain between our study and other studies and countries may be attributed to differences in study designs and population characteristics. The observed discrepancies in prevalence rates can be attributed to variations in both the methodological approaches and contextual factors specific to Saudi Arabia. Differences in sample size, recruitment strategies, data collection methods, and statistical analyses employed across studies are likely contributors. Additionally, factors like healthcare access and utilization patterns, health status, region of residence, and income level within Saudi Arabia may have influenced these methodological choices and further contributed to the observed discrepancies [19].

In this study, most patients who experienced pain were female (65.8%). The trend of females exhibiting a higher prevalence of pain than males is widespread and has been demonstrated domestically [14,15] and globally [20,21]. Numerous hypotheses regarding hormonal, reproductive, and genetic factors have been proposed to explain these sex differences. Moreover, studies have suggested that females may have lower pain thresholds, resulting in greater exposure to pain and less tolerance to stimuli. However, these hypotheses are not supported by sufficient evidence, and studies investigating this topic have failed to identify the anatomical or physiological factors [21].

Our study revealed that the most commonly reported pain region was the back (38.6%), followed by the neck (16.6%), shoulders (15.3%), and knees (14.8%). Our results are similar to those of other studies performed in Saudi Arabia that reported back pain, with a prevalence of 30% [11] and 25.2% [15]. Globally, the prevalence of LBP was estimated to be higher in high-income countries (30%) compared to low-income countries (18.2%) [22]. Differences in the prevalence of pain in other body regions were evident. For example, the second and third most common regions of pain in a study by El-Metwally et al. [11] were the abdomen (26%) and head (13%) and were the lower limbs (21.9%) and head (12.7%) in a study by Almalki et al. [15]. Our study revealed a higher prevalence of inpatient admissions among patients with back or knee pain compared to those with neck or shoulder pain. This finding might be explained by the availability of specialized resources for back and knee surgeries at KFHU. The hospital employed three surgeons for lumbar spine and three for knee, whereas only one surgeon treated shoulder pain, and there were no dedicated surgeons for neck pain. Additionally, a key performance indicators study by the KFHU physical therapy department suggested that patients with neck pain experienced better treatment outcomes than those with LBP, potentially reducing the need for surgical intervention and inpatient admission.

In the current study, age was moderately associated with pain region. Older patients exhibited more prevalent pain in the shoulder than in the neck, back, and knee and more pain in the knee than in the back. It is important to acknowledge that the age difference between the groups according to pain region did not exceed 4.5 years (back, 50.3 ± 14.3; neck, 51.6 ± 12.4; shoulder, 54.8 ± 14.4; and knee, 52.2 ± 16.5 years). Patients of 50–69 years of age had the highest prevalence of pain across all body regions. The positive relationship between age and pain prevalence is well documented in domestic [15] and international research [23]. A study conducted in the United States on the burden of musculoskeletal diseases found that shoulder pain was the second most common joint site for chronic pain, after knee pain. Of individuals aged 18 and older, 22.3 million reported experiencing shoulder pain, with similar rates observed across the age group of 45 and older [24]. The higher prevalence of pain among the elderly could be attributed to changes in structures related to pain processing and other changes that alter the transmission and processing of pain stimuli. These changes involve peripheral nerves and receptors, spinal cord and descending modulating systems, and brain and supraspinal structures [23]. Our study demonstrates associations, not causation, between the investigated variables as mentioned above. Establishing causal relationships was not within the scope of the study because of observational design [25]. Potential confounding variables might influence the observed associations. This might involve demographic factors, clinical factors, and methodological factors [26,27]. These variables could potentially bias the observed associations, even though the study may not have explicitly controlled for them.

### Limitations

This study had several limitations. A major limitation was that the EHR system lacked data on crucial pain descriptors, including severity, type (e.g., nociceptive, neuropathic, and nociplastic), and diagnostic classifications. Additionally, vital demographic data such as chronicity of pain, body mass index, smoking history, and occupation were missing. Notably, the diagnostic terms for pain and disorders varied across the dataset, either being missing entirely or entered inconsistently by different providers. We recognize potential impacts of diagnostic term discrepancies on our study outcomes. For instance, inaccurate diagnoses in the EHR could lead to the misclassification of participants, potentially influencing the observed associations between variables, and consequently, lead to underestimating or overestimating the results of the study. Moreover, inconsistencies in diagnostic coding could limit the generalizability of our findings to broader populations. Consequently, we recommend recoding the diagnostic terms into a unified system, such as the International Classification of Diseases, to enhance data consistency and analysis. Employing data validation procedures, such as automated checks and manual chart review, can also help identify and correct errors in diagnostic terms before analysis. In addition, engaging with healthcare providers and information technology staff responsible for the EHR system can facilitate the implementation of data quality improvements and promote standardized coding practices. Another limitation of the study is that it was conducted in a single center and region of Saudi Arabia. The generalizability of findings to the entire country remains limited. We acknowledge the potential for regional variations in the phenomenon under study and recognize that our sample may not be fully representative of the national population. Retrospective designs like our study, while valuable for understanding past trends, inherently struggle to capture the details of pain experiences. These limitations include reliance on existing data, potential recall bias, lack of standardized pain assessment tools, limited information about contributing factors, and difficulty establishing causality. Despite these limitations, our findings contribute valuable insights, aligning with other domestic and international studies.

## 5. Conclusions

Results of this study highlight the prevalence of pain and its association with age and sex in adult patients referred for physical therapy in Saudi Arabia over a 5-year period. Approximately 26.0% (*n* = 7426) of these patients experienced pain in different regions of the body, with a higher prevalence in the back, neck, shoulders, and knees. Moderate relationships were found between age and pain region(s) and between sex and pain region(s). Female patients and older patients appeared to exhibit a higher prevalence of pain. Future research through multicenter studies with diverse regional representation would be crucial for achieving a broader applicability of findings.

## Figures and Tables

**Table 1:** Comparison of demographic characteristics between patients with and without pain.

	Pain Patients (*n* = 7426)	Non-Pain Patients (*n* = 21,195)	Total (*n* = 28,621)
Age (years)	51.4 ± 15.0	52.4 ± 18.1	52.2 ± 17.4
*Gender*			
Female	4884 (65.8%)	11,292 (53.3%)	16,002 (55.9%)
Male	2542 (34.2%)	9903 (46.7%)	12,619 (44.1%)
*Nationality*			
Saudi	6291 (84.7%)	17,609 (83.1%)	23,923 (83.6%)
Non-Saudi	1135 (15.3%)	3586 (16.9%)	4698 (16.4%)
*Referral type*			
Outpatient	6611 (89%)	14,182 (66.9%)	20,801 (72.7%)
Inpatient	815 (11%)	7013 (33.1%)	7820 (27.3%)
*Referring service*			
Orthopedics	1347 (18.1%)	4588 (21.6%)	5948 (20.8%)
Neurosurgery	374 (5.0%)	1094 (5.2%)	1464 (5.1%)
Neurology	225 (3.0%)	1503 (7.1%)	1730 (6.0%)
Internal medicine	127 (1.7%)	1283 (6.1%)	1402 (4.9%)
Others (not specified) *	5162 (69.5%)	10,982 (51.8%)	16,124 (56.3)
Others (specified) †	190 (2.6%)	1745 (8.2%)	1953 (6.8%)

Data are presented as frequency (%) except age, which is shown as mean ± SD. * Referring service was not specified on the system. † These services included cardiology, pediatrics, pediatric surgery, general surgery, obstetrics and gynecology, urology, nephrology, pulmonary/ICU, thoracic/cardiac, gastroenterology, plastic surgery, ENT, chest surgery, hematology/oncology, orthodontic, anesthesia, critical care, psychology, dental clinic, hepatology, ophthalmology, rheumatology/allergy, and referrals from outside the hospital, either from the Saudi Ministry of Health or other facilities.

**Table 2:** Location of pain by anatomical body region (*n* = 7426).

Pain Region	Frequency	Percentage (%)
Head/face	36	0.5
Neck	977	13.2
Chest	121	1.6
Shoulder	901	12.1
Elbow	41	0.6
Wrist/hand	153	2.1
Abdomen	83	1.1
Back *	2279	30.7
Pelvis	38	0.5
Hip	121	1.6
Knee	873	11.8
Ankle/foot	272	3.7
Other ^$^	1531	20.6

Some patients may have pain in more than one region (See text). * Low back pain represents 581 (25.5%) of these cases. ^$^ Majority of these cases were not specified to a body region, whereas 169 and 125 cases were related to leg and arm, respectively.

**Table 3:** Characteristics of patients (*n* = 5894) in the four most common pain regions.

	Back (*n* = 2278 (38.6%))	Neck (*n* = 977 (16.6%))	Shoulder (*n* = 901 (15.3%))	Knee (*n* = 873 (14.8%))
*Age (years)*	50.3 ± 14.3	51.6 ± 12.4	54.8 ± 14.4	52.2 ± 16.5
*Age groups (years)*				
<30	180 (7.9)	42 (4.3)	50 (5.5)	91 (10.4)
30–49	873 (38.3)	363 (37.2)	241 (26.7)	283 (32.4)
50–69	1046 (45.9)	501 (51.3)	473 (52.5)	372 (42.6)
≥70	179 (7.9)	71 (7.3)	137 (15.2)	127 (14.5)
*Gender*				
Male	811 (35.6)	183 (18.7)	211 (23.4)	282 (32.3)
Female	1467 (64.4)	794 (81.2)	690 (76.6)	591 (67.7)
*Nationality*				
Saudi	1941 (85.2)	829 (84.9)	776 (86.1)	736 (84.3)
Non-Saudi	337 (14.8)	148 (15.1)	125 (13.9)	137 (15.7)
*Care type*				
Outpatient	2070 (90.9)	965 (98.8)	887 (98.4)	754 (86.4)
Inpatient	208 (9.1)	12 (1.2)	14 (1.6)	119 (13.6)

Data are presented as frequency (percentage), except age (mean ± SD). Percentage represents % of patients with pain out of total people at a specific region.

## Data Availability

The data that support the findings of this study are available on request from the corresponding author. The data are not publicly available due to their containing information that could compromise the privacy of the hospital.
